# Plant-Based Diet: A Potential Intervention for Heart Failure

**DOI:** 10.7759/cureus.8282

**Published:** 2020-05-25

**Authors:** Faris A Alasmre, Hammam A Alotaibi

**Affiliations:** 1 Family and Community Medicine, King Khalid University, Abha, SAU; 2 Research, Prince Sultan Military Medical City, Riyadh, SAU

**Keywords:** plant-based diet, interventional study, heart failure

## Abstract

Heart failure is a disease that increases the likelihood of morbidity and mortality with an increased direct and indirect cost to the healthcare system. The role of diet in the development, progression and treatment of heart failure is being studied with growing interest. The objective of this study is to assess the relationship between heart failure and selected heart failure risk factors: hyperlipidemia, hypertension, diabetes mellitus and plant-based diets as a clinical intervention. The search yielded 416 published articles, among them are three studies that used a plant-based diet as an intervention for heart failure. All interventional studies showed that plant-based diets have a positive impact on heart failure in terms of improved ejection fraction and positive cardiac muscle remodelling.

## Introduction and background

Heart failure (HF) is a disease that increases the risk of morbidity and mortality alongside an increased direct and indirect costs to the healthcare system. The role of diet in the development, progression and treatment of heart failure is being studied with growing interest [1].

The disease is defined by the heart’s inability to pump enough blood to provide the metabolic requirements of the body. It can present with preserved or reduced left ventricular ejection fraction [1]. Risk factors of HF are multifaceted and are affected significantly by diet; they include obesity, hyperlipidemia, hypertension, and diabetes mellitus [2]. Heart failure is one of the most common causes of morbidity and mortality in the world. The disease prevalence is more than 5.5 million in the US alone and 23 million globally. Annually, in the US alone, more than 550,000 are diagnosed with HF, half of them die within the first five years [3,4].

Plant‐based diets are a group of dietary choices that focus on increased intake of plant foods and reduce animal-based foods. Vegetarian diets, a type of plant‐based diet, emphasize the restriction of different types of animal-based food choices like meat, poultry, or fish, and have been associated with a reduced risk of cardiovascular risk factors, such as obesity, hypertension, diabetes mellitus, and ischemic heart disease [5,6]. Epidemiological studies in the past 10 years alone showed an up to 81% reduction in heart failure incidence in groups adhering to a healthy lifestyle with regular exercise, healthy dietary choice and normal BMI. Thus, the role of nutrition has been deemed an important one in studying cardiovascular disease [7,8].

The objective of this study is to assess the relationship between heart failure and plant-based diets as a clinical intervention. There is mounting evidence in the observational literature with respect to the health benefits of plant-based diets with limited emphasis on clinical interventions, this study focuses on clinical intervention as single or augmented therapy for heart failure. Moreover, this research focuses on summarizing the published evidence on the effects of plant-based diets on heart failure to provide a clearer understanding of the role nutrition can play in the treatment of the disease.

## Review

This is a literature summary designed to qualitatively report relevant findings on the effects of a plant-based diet in heart failure patients or patients with selected risk factors of heart failure in clinical trials conducted from 2000 to March 2020. The objective of this study is to summarize the relevant interventional literature on plant-based diets and heart failure. All clinical trials published since 2000 until March 2020 were reviewed. Databases used for the search include PubMed, Medline, the Cochrane collaboration. Key words were: “Vegetarian”, “vegan”, “plant-based diet” and “heart failure”. The search yielded 416 articles. Limitations used were: Published after 2000, English language and clinical trials. Results were excluded if written before 2000, written in languages other than English and if the sample under study is cardiovascular disease other than heart failure. Studies that did not address the question directly were also excluded.

The search yielded 416 published articles, among them, three studies used a plant-based diet as an intervention for heart failure. Findings are summarized in Table 1.

**Table 1:** Summary of included studies BMI: Body mass index; CHD: Coronary heart disease; CHF: Congestive heart failure; cMRI: Cardiac magnetic resonance imaging; EF: Ejection fraction; LDL: Low density lipoprotein; LVEF: Left ventricular ejection fraction; LVSD: Left ventricular systolic dysfunction; n: number of patients.

Study	Design	Diet type	Effect	Result	Comments
Pischke et al., 2007 [9]	Interventional study for CHD (Analysis of HF) n = 46	Plant-based diet	LVEF impairment	Improved exercise tolerance and improved HF risk factors.	Three months of plant-based diet significantly decreased BMI, LDL, and significantly improved angina frequency and severity and reduced physical limitations.
Choi et al., 2017 [10]	Interventional study n = 1	Whole food plant-based diet	HF (LVSD) EF = 35%	Left ventricular ejection fraction increased to 50%.	60 days of whole food plant-based diet improved EF significantly.
Najjar and Montgomery, 2019 [11]	Interventional study n = 3	Plant-based diet	CHF	cMRI: 92% improvement in EF, 21% reduction in left ventricular mass, 62% increase in stroke volume, 17% increase in cardiac output.	Plant-based diet for mean of 79 days significantly improved symptoms of heart failure.

Pischke et al. in 2007 conducted an analysis on the effects of plant-based diets on ejection fraction in patients with heart disease. In 46 patients with left ventricular ejection fraction impairment, all patients were put on a plant-based diet for three months and were followed throughout. They found that all heart failure risk factors have improved during that period along with exercise tolerance. This finding shows that plant-based diets have a direct positive impact on clinical outcomes of heart failure in this sample group [9].

In 2017, Choi et al. reported one heart failure case with ejection fraction of 35% and left ventricular systolic dysfunction. This patient was on plant-based diet for two months. The outcome of the intervention showed major changes in ejection fraction as it normalized to 50% and other heart failure risk factors improved significantly over that period [10].

In 2019, Najjar and Montgomery reported three cases of heart failure patients who were put on plant-based diets for an average of 79 days. All patients showed a major improvement in heart failure risk factors and blood markers, and substantial changes in heart structure and function where ejection fraction improved by 92%, LV mass reduced by 21%, stroke-volume increased by 62% and overall, cardiac output increased by 17% in the patient group [11].

Currently, the observational literature indicates that there might be a growing observed positive impact plant-based diets have on heart failure development and progression. Current research indicates that antioxidants, micronutrients and aversion to high fat nutritional choices are associated with reduction in heart failure incidence and morbidity. One potential explanation is the differing factors related to reduction of oxidative stress and inflammation [12]. In terms of clinical interventional studies, the limitation of available research is much more visible. There are a few interventional studies on the effects of plant-based diets as an augmented treatment for heart failure. The published literature today consists of analysis of interventional study and two case reports totalling four patients when combined. The observed findings show a clear and direct positive impact on ejection fraction and cardiac muscle remodelling as shown in Table 1. These findings indicate that plant-based diets have the potential to be a powerful augmented therapy to heart failure treatment protocols and warrant further investigation.

This study has the following limitations: first, the small sample size of the case reports cannot be used to generalize the findings to all patients with heart failure and should be seen as a limited finding that points only to potential benefit. Larger studies are necessary to provide statistical power and more rigorous design to reduce the effects of potential confounders to a minimum. Second, plant-based diets incorporate diverse effective components such as varying micronutrients, different phytochemicals and antioxidants, along with reduction in saturated fat and dietary salt among other factors that have the potential to improve symptoms of heart failure. This means that such interventional studies included in this review although promising cannot establish the precise cause of improvement in heart failure patients under study. Finally, causation cannot be determined from the body of the literature whether observational or interventional due to the lack of rigorous interventional design with a sample size enough to establish statistical significance. Thus, a larger study with interventional design is needed.

## Conclusions

Plant-based diets show promising results in heart failure patients and should be viewed as an important adjunct to standard treatment. A number of small studies in the past two decades show a consistent positive clinical and risk factor improvements in patients with heart failure. These findings, although in small samples, can lead the way for more interventional studies with more rigorous design to shed more light on the effects of plant-based diet on heart failure as a clinical intervention.
